# Retrospective analysis of risk factors for distant metastasis of early-onset gastric cancer during the perioperative period

**DOI:** 10.3389/fonc.2023.1003977

**Published:** 2023-02-03

**Authors:** Bo Bi, Guo-fei Deng, Yun-min Duan, Zhi-jian Huang, Xiao-yan Chen, Chang-hua Zhang, Yu-long He

**Affiliations:** ^1^ Guangdong Provincial Key Laboratory of Digestive Cancer Research, The Seventh Affiliated Hospital of Sun Yat-sen University, Shenzhen, Guangdong, China; ^2^ School of Nursing, Shandong First Medical University Shandong Academy of Medical Sciences, Jinan, Shandong, China; ^3^ Department of Gastrointestinal Surgery, The First Affiliated Hospital of Sun Yat-Sen University, Guangzhou, Guangdong, China

**Keywords:** gastric cancer, metastasis, early-onset, prediction model, nomogram

## Abstract

**Background:**

Although the overall global incidence of gastric cancer has been declining, the number of new cases in people under the age of 50 is increasing, which is related to metastasis, late pathological stages, and poor prognosis. There is a scarcity of large-scale studies to evaluate and predict distant metastasis in patients with early-onset gastric cancer.

**Methods:**

From January 2010 to December 2019, data on early-onset GC patients undergoing surgery were gathered from the Surveillance, Epidemiology, and End Results (SEER) database. We investigated the independent risk factors for distant metastasis in patients with early-onset gastric cancer. Based on these risk factors, we developed a nomogram to predict distant metastasis. The model underwent internal validation on the test set and external validation on 205 patients from the First Affiliated Hospital of Sun Yat-sen University and the seventh Affiliated Hospital of Sun Yat-sen University. The novel nomogram model was then evaluated using the receiver operating characteristic (ROC) curve, calibration, the area under the curve (AUC), and decision curve analysis (DCA). The training set nomogram score was used to classify the different risk clusters of distant metastasis.

**Results:**

Our study enrolled 2217 patients after establishing the inclusion and exclusion criteria, with 1873 having no distant metastasis and 344 having distant metastasis. The tumor size, total lymph nodes, whether or not receiving radiotherapy and chemotherapy, T stage, and N stage were significant predictors of advanced distant metastasis (*p* < 0.05). The AUC of the ROC analysis demonstrated our model’s high accuracy. Simultaneously, the prediction model shows high stability and clinical practicability in the calibration curve and DCA analysis.

**Conclusions:**

We developed an innovative nomogram containing clinical and pathological characteristics to predict distant metastasis in patients younger than 50 years old with gastric cancer. The tool can alert clinicians about distant metastasis and help them develop more effective clinical treatment plans.

## Introduction

Despite regular screening and advanced treatment effectively reducing gastric cancer (GC) mortality, GC still ranks fourth in the cause of cancer deaths worldwide (1). The risk factors closely related to gastric cancer include Helicobacter pylori infection, smoking, drinking, low income, high salt intake, family tendency, previous history of gastric surgery, and pernicious anemia (2). Under the influence of various environmental and genetic factors, gastric cancer mainly occurs in middle-aged and elderly patients aged 50-70. However, it is relatively uncommon in the younger population (under 50 years old) (2, 3), which is known as early-onset GC (4, 5). The Surveillance Epidemiology and End Results (SEER) program recently revealed a significant rise in the incidence of early-onset GC in both female and male patients (6). Young patients are more likely to ignore clinical manifestations because the early symptoms are not obvious. Meanwhile, studies have shown that younger patients in various cancers have more distant metastasis (DM) and poorer outcomes than older patients (7, 8). Nevertheless, many GC patients are typically diagnosed at an advanced or metastatic stage, indicating a dismal 5-year survival rate of less than 30% (9). In early-onset GC, it is critical to accurately evaluate and predict metastasis status for treatment choice and prognostic assessment.

This study sought to investigate the clinicopathological traits and prognostic factors of DM patients with early-onset GC who were under 50 years old. In this retrospective analysis, 2217 patients from the SEER database diagnosed with early-onset GC were assigned randomly to the training and test sets. Using logistic regression analysis, we discovered the clinicopathological factors influencing the DM of early-onset GC. Then, utilizing these risk factors, we developed a nomogram to optimize the precision of predicting DM in early-onset GC. Our center’s early-onset GC patients served as validation subjects for the model. The findings indicate that our model can accurately predict the DM of early-onset GC.

## Materials and methods

### Clinical information

**Figure 1:**
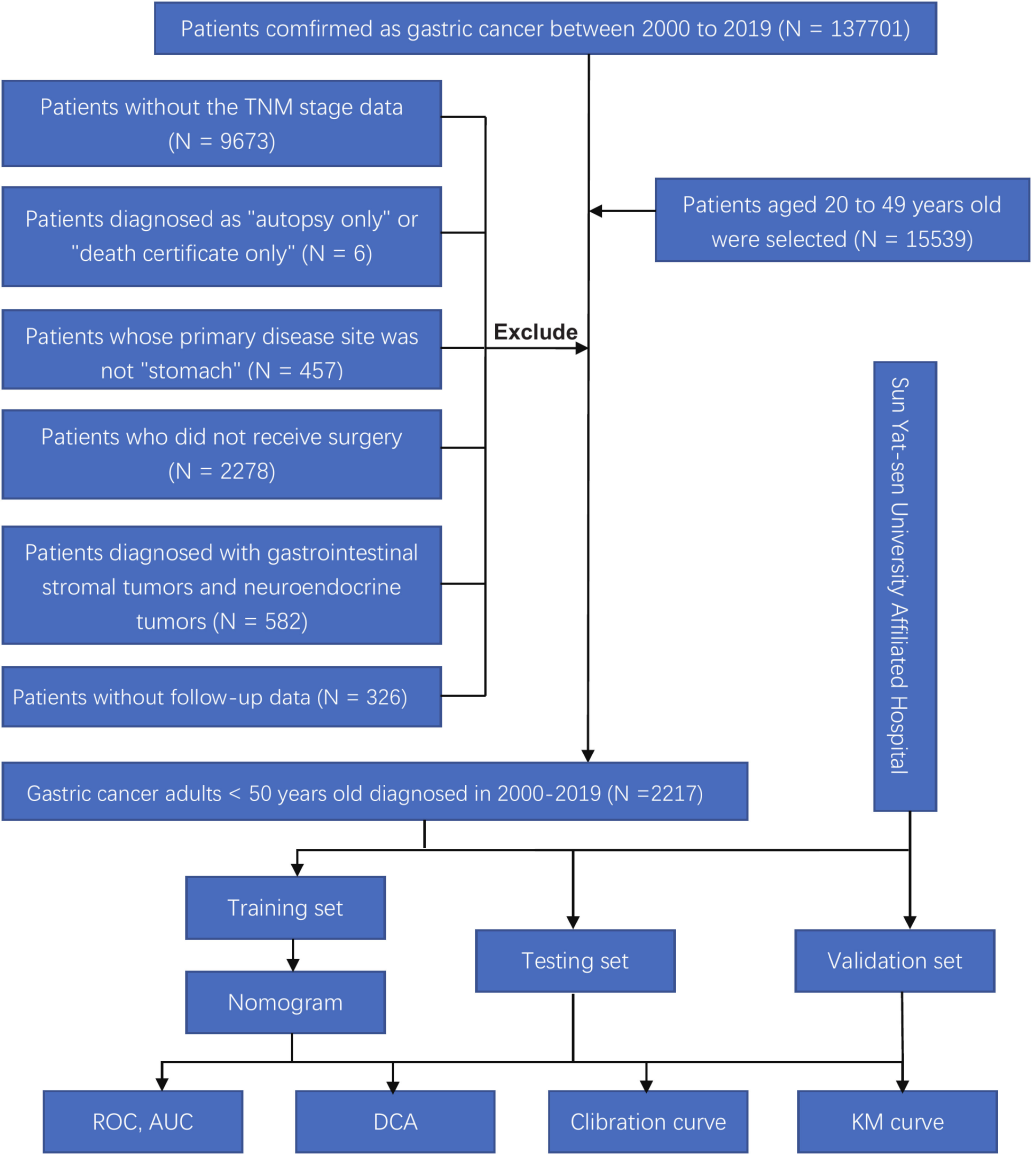
Flow chart of this study. ROC, receiver operating characteristic; AUC, area under the curve; DCA, and decision curve analysis; KM curve, Kaplan-Meier curve.

We downloaded and filtered the clinical data of GC individuals diagnosed between 2010 and 2019 using the software seer*stata 8.4.0. The following criteria were used to exclude patients: (1) Patients with carcinoma *in situ* or without TNM staging data, according to the 7th American Joint Commission on Cancer (AJCC). (2) Patients who have been diagnosed with “autopsy only” or “death certificate only.” (3) Patients whose primary disease site is not the stomach (4) Patients who have not received surgery (5) Patients suffering from neuroendocrine and gastrointestinal stromal tumors (6) Patients without follow-up information. The flow chart of this retrospective study was shown in **Figure 1**. We analyzed and compared the age, gender, race, marital status, location of the tumor, tumor differentiation, tumor stage, tumor diameter, chemo and radiation, the total number of lymph nodes acquired, overall survival (OS), and cancer-specific survival (CSS) between groups with different metastatic status.

After identifying 2217 patients, they were arbitrarily split into a training and an internal test set in the proportion of 6:4. External validation of the model was performed on 205 early-onset GC patients from the First Affiliated Hospital of Sun Yat-sen University and the seventh Affiliated Hospital of Sun Yat-sen University. The ethical committees of the first and seventh Affiliated Hospital of Sun Yat-sen University approved this study.

### Risk factor analysis and prediction model development

We investigated the risk factors for DM in early-onset GC patients. First, we utilized the Kaplan-Meier method to assess the impact of DM on the OS and CSS of the selected cases in the database and our follow-up cases. The chi-square test and logistic regression were then performed to investigate the potential risk factors for DM in patients with early-onset GC. After that, in the training set, a logistic regression nomogram was established and validated in the test and validation sets to predict the probability of DM. By plotting the ROC curve, we measured the model’s sensitivity and specificity to estimate patients’ OS and then obtained the AUC to evaluate the model’s accuracy. The calibration curve was then drawn to assess the reliability between the expected and observed outcomes of the nomogram. Meanwhile, we evaluated the clinical efficacy of the model using DCA curves. Next, we calculated the scores of all the patients in the training sets using the nomogram, classified them as low or high risk, and examined the OS of patients in each risk group. Finally, we applied the scoring system to our external validation set to test the clinical applicability of the model further and observed whether the scoring system was applicable in clinical practice in the inter-group comparison between subgroups with different clinical-pathological characteristics.

### Statistical evaluation

All statistical analyses in this study were conducted with R version 4.1.0. Categorical variables were represented numerically with percentages. The Chi-square test was utilized to compare variables of pathological characteristics. The survival of patients with or without DM was analyzed using Kaplan-Meier curves. To identify the independent risk factors of DM, Variables with a p-value < 0.1 in the univariate logistic regression analysis were included in the multivariate logistic regression analysis to determine the risk factors for DM in early-onset GC. The nomogram and calibration curve are generated with the R package “rms.” The “pROC” package was used to draw the ROC curve and obtain the specific value of AUC. The “rmda” package was used to plot a DCA curve to describe the nomogram model’s clinical performance compared to the single factors. A p-value < 0.05 (two‐tailed) was deemed statistically significant in all statistical calculations.

## Results

### Baseline characteristics of patients

**Figure 2:**
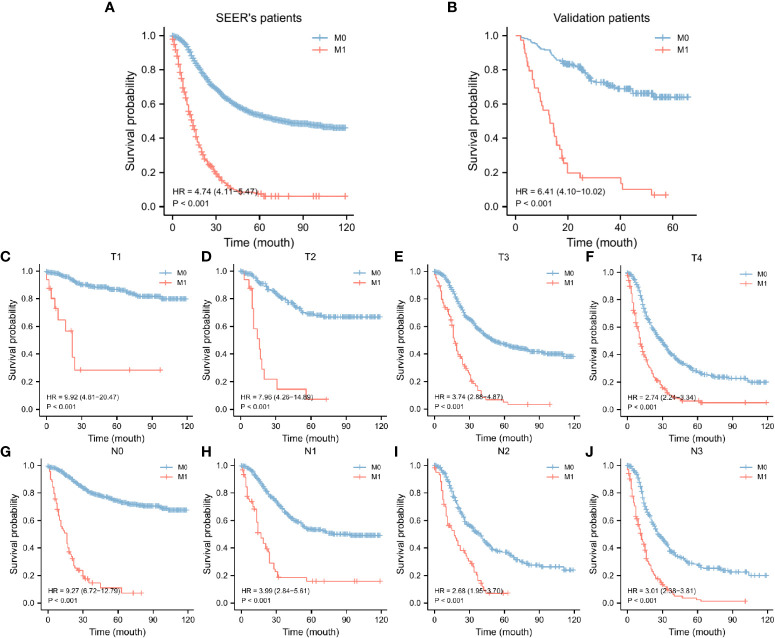
KM curves of OS for GC in the SEER data set **(A)** and our validation set **(B)**, KM curves of OS for GC in the T stages **(C-F)** and N stages **(G-J)**. SEER, Surveillance, Epidemiology, and End Results database; OS, overall survival; GC, gastric cancer; KM curve, Kaplan-Meier curve.

In the SEER database, 2217 early-onset GC patients who had undergone surgery were screened, 344 of whom developed DM. **Table 1** summarizes the general characteristics of all patients. Age, gender, primary site, tumor size, whether radiation or chemotherapy was utilized, and the total number of lymph nodes removed during surgery varied statistically between the two groups (*p* < 0.05). Following that, patients were randomly assigned to two groups: a training group of 1330 individuals and an internal test group of 887 individuals. As shown in **Table 2**, demographic and pathological disparity were not statistically significant. Then, we gathered 205 early-onset GC patients from the First Affiliated Hospital of Sun Yat-sen University and the seventh Affiliated Hospital of Sun Yat-sen University as an external validation set to ensure the study’s universality. **Table S1** shows the basic features of these patients, 39 of whom had DM. The SEER database and our validation cohort exhibited a shorter OS for patients with DM, as indicated by the Kaplan Meier curve (**Figures 2A, B**). The DM patients also had significantly lower CCS (**Figure S1**). At the same time, we examined the effect of different distant metastasis states on the prognosis of different T (**Figures 2C–F**) and N (**Figures 2G–J**) stages.

**Table 1:** Demographic data and clinical characteristics.

	M0	M1	*p*
n	1873	344	
Age (%)			0.001
20-29	103 (5.5)	26 (7.6)	
30-39	443 (23.7)	108 (31.4)	
40-49	1327 (70.8)	210 (61.0)	
Sex = Male/Female (%)	1051/822 (56.1/43.9)	161/183 (46.8/53.2)	0.002
Marital (%)			0.182
Married	1110 (59.3)	201 (58.4)	
Single	663 (35.4)	132 (38.4)	
Unknown	100 (5.3)	11 (3.2)	
Race (%)			0.679
Black	228 (12.2)	47 (13.7)	
White	1265 (67.5)	225 (65.4)	
Others	380 (20.3)	72 (20.9)	
Primary_Site (%)			<0.001
Lower	488 (26.1)	102 (29.7)	
Middle	241 (12.9)	32 (9.3)	
Upper	544 (29.0)	52 (15.1)	
not_exactly	600 (32.0)	158 (45.9)	
Grade (%)			0.069
Grade I	51 (2.7)	5 (1.5)	
Grade II	246 (13.1)	30 (8.7)	
Grade III	1183 (63.2)	240 (69.8)	
Grade IV	44 (2.3)	9 (2.6)	
GX	349 (18.6)	60 (17.4)	
Stage (%)			<0.001
I	448 (23.9)	0 (0.0)	
II	498 (26.6)	0 (0.0)	
III	833 (44.5)	0 (0.0)	
IV	7 (0.4)	344 (100.0)	
X	87 (4.6)	0 (0.0)	
T (%)			<0.001
T1	405 (21.6)	16 (4.7)	
T2	214 (11.4)	16 (4.7)	
T3	713 (38.1)	85 (24.7)	
T4	467 (24.9)	195 (56.7)	
Tx	74 (4.0)	32 (9.3)	
N (%)			<0.001
N0	731 (39.0)	69 (20.1)	
N1	415 (22.2)	59 (17.2)	
N2	317 (16.9)	61 (17.7)	
N3	365 (19.5)	131 (38.1)	
NX	45 (2.4)	24 (7.0)	
M = M0/M1 (%)	1873/0 (100.0/0.0)	0/344 (0.0/100.0)	<0.001
Radiation = No/Yes (%)	1130/743 (60.3/39.7)	277/67 (80.5/19.5)	<0.001
Chemotherapy = No/Unknown/Yes (%)	467/1406 (24.9/75.1)	62/282 (18.0/82.0)	0.007
Tumor_Size (%)			<0.001
<50	926 (61.6)	96 (38.7)	
50-100	500 (33.2)	108 (43.5)	
>100	78 (5.2)	44 (17.7)	
Total_nodes (%)			<0.001
<8	303 (16.2)	97 (28.2)	
8~16	483 (25.8)	76 (22.1)	
17-30	665 (35.5)	106 (30.8)	
>30	375 (20.0)	46 (13.4)	
Unkown	47 (2.5)	19 (5.5)	

**Table 2:** Baseline clinical characteristics of patients in training set and validation set.

	Training	Validation	*p*
n	1330	887	
Age (%)			0.733
20-29	80 (6.0)	49 (5.5)	
30-39	336 (25.3)	215 (24.2)	
40-49	914 (68.7)	623 (70.2)	
Sex = Male/Female (%)	716/614 (53.8/46.2)	496/391 (55.9/44.1)	0.356
Marital (%)			0.331
Married	803 (60.4)	508 (57.3)	
Single	461 (34.7)	334 (37.7)	
Unknown	66 (5.0)	45 (5.1)	
Race (%)			0.233
Black	163 (12.3)	112 (12.6)	
White	880 (66.2)	610 (68.8)	
Others	287 (21.6)	165 (18.6)	
Primary_Site (%)			0.683
Lower	345 (25.9)	245 (27.6)	
Middle	160 (12.0)	113 (12.7)	
Upper	359 (27.0)	237 (26.7)	
not_exactly	466 (35.0)	292 (32.9)	
Grade (%)			0.214
Grade I	37 (2.8)	19 (2.1)	
Grade II	170 (12.8)	106 (12.0)	
Grade III	840 (63.2)	583 (65.7)	
Grade IV	39 (2.9)	14 (1.6)	
GX	244 (18.3)	165 (18.6)	
Stage (%)			0.667
I	260 (19.5)	188 (21.2)	
II	297 (22.3)	201 (22.7)	
III	504 (37.9)	329 (37.1)	
IV	220 (16.5)	131 (14.8)	
X	49 (3.7)	38 (4.3)	
T (%)			0.717
T1	247 (18.6)	174 (19.6)	
T2	139 (10.5)	91 (10.3)	
T3	489 (36.8)	309 (34.8)	
T4	397 (29.8)	265 (29.9)	
TX	58 (4.4)	48 (5.4)	
N (%)			0.182
N0	476 (35.8)	324 (36.5)	
N1	287 (21.6)	187 (21.1)	
N2	241 (18.1)	137 (15.4)	
N3	280 (21.1)	216 (24.4)	
NX	46 (3.5)	23 (2.6)	
M = M0/M1 (%)	1115/215 (83.8/16.2)	758/129 (85.5/14.5)	0.33
Radiation = No/Yes (%)	844/486 (63.5/36.5)	563/324 (63.5/36.5)	1
Chemotherapy = No/Unknown/Yes (%)	311/1019 (23.4/76.6)	218/669 (24.6/75.4)	0.552
Tumor_Size (%)			0.433
<50	609 (45.8)	413 (46.6)	
50-100	380 (28.6)	228 (25.7)	
>100	73 (5.5)	49 (5.5)	
not_exactly	268 (20.2)	197 (22.2)	
Total_nodes (%)			0.16
<8	249 (18.7)	151 (17.0)	
8~16	313 (23.5)	246 (27.7)	
17-30	474 (35.6)	297 (33.5)	
>30	250 (18.8)	171 (19.3)	
Unkown	44 (3.3)	22 (2.5)	

### Independent risk factors of DM in patients with early-onset GC

**Figure 3:**
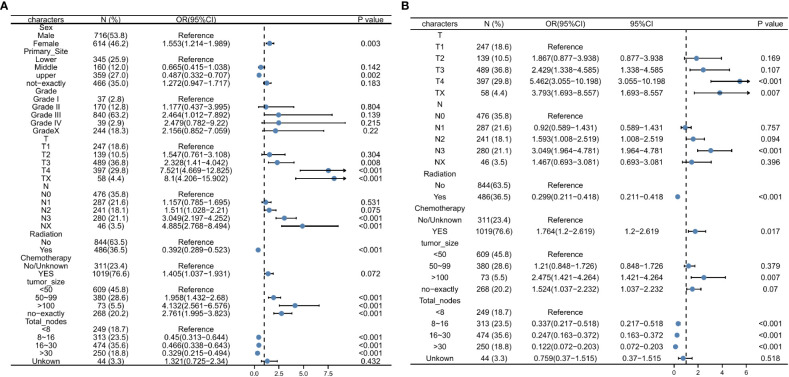
Univariate logistic regression analysis for predicting the DM **(A)**, Multivariate logistic regression analysis for predicting the DM **(B)**. DM, distant metastasis.

Univariate logistic regression analysis of the training set revealed that DM was significantly correlated with sex, primary site, tumor size, number of lymph nodes cleaned during surgery, and whether chemotherapy or radiation was used (**Table 3**; **Figure 3A**). These variables were considered in the subsequent multivariate logistic regression analysis (**Table 3**; **Figure 3B**). T4 patients (OR = 5.462, 95% CI = 3.055-10.198, *p* < 0.001) and Tx patients (OR = 3.793, 95% CI = 1.693-8.557, *p* = 0.007) were more likely to develop DM than T1 patients. Patients in the N3 stage were more likely than those in the N0 stage to have DM (OR = 3.049, 95% CI = 1.964-4.781, *p <* 0.001). Patients with tumors larger than 100 mm had an increased risk of developing DM (OR = 2.47, 95% CI = 1.421-4.264, *p* = 0.007) compared to those with tumors smaller than 50 mm. If more lymph nodes are removed during surgery, patients with GC have a lower risk of developing DM. Intriguingly, we discovered that radiotherapy in conjunction with a surgical procedure (OR =0.299, 95% CI = 0.211-0.418, *p* = 0.001) was a protective factor for DM of early-onset GC. Additionally, chemotherapy treatment was a significant risk factor (OR = 1.764, 95% CI = 1.2-2.619, *p* = 0.017).

**Table 3:** Univariate and multivariate logistic regression analyses of distant metastasis in early-onset gastric cancer from training set.

Variables	Univariate analysis			Multivariate analysis		
	OR	95%CI	*p*	OR	95%CI	*p*
Age, years						
20-29	Reference					
30-39	0.978	0.594-1.662	0.942			
40-49	0.681	0.426-1.13	0.193			
Sex						
Male	Reference			Reference		
Female	1.553	1.214-1.989	0.003	0.084	1.016-1.785	0.083
Marital_status						
Married	Reference					
Single	1.238	0.958-1.595	0.168			
Unknown	0.548	0.247-1.066	0.17			
Race						
Black	Reference					
White	0.997	0.687-1.481	0.989			
Others	1.085	0.705-1.691	0.759			
Primary_Site						
Lower	Reference			Reference		
Middle	0.665	0.415-1.038	0.142	0.619	0.367-1.019	0.121
upper	0.487	0.332-0.707	0.002	0.933	0.608-1.424	0.789
not-exactly	1.272	0.947-1.717	0.183	1.06	0.761-1.482	0.773
Grade						
Grade I	Reference					
Grade II	1.177	0.437-3.995	0.804			
Grade III	2.464	1.012-7.892	0.139			
Grade IV	2.479	0.782-9.22	0.215			
GradeX	2.156	0.852-7.059	0.22			
T						
T1	Reference			Reference		
T2	1.547	0.761-3.108	0.304	1.867	0.877-3.938	0.169
T3	2.328	1.41-4.042	0.008	2.429	1.338-4.585	0.107
T4	7.521	4.669-12.825	<0.001	5.462	3.055-10.198	<0.001
TX	8.1	4.206-15.902	<0.001	3.793	1.693-8.557	0.007
N						
N0	Reference			Reference		
N1	1.157	0.785-1.695	0.531	0.92	0.589-1.431	0.757
N2	1.511	1.028-2.21	0.075	1.593	1.008-2.519	0.094
N3	3.049	2.197-4.252	<0.001	3.049	1.964-4.781	<0.001
NX	4.885	2.768-8.494	<0.001	1.467	0.693-3.081	0.396
Radiation						
No	Reference			Reference		
Yes	0.392	0.289-0.523	<0.001	0.299	0.211-0.418	<0.001
Chemotherapy						
No/Unknown	Reference			Reference		
YES	1.405	1.037-1.931	0.072	1.764	1.2-2.619	0.017
tumor_size						
<50	Reference			Reference		
50-99	1.958	1.432-2.68	<0.001	1.21	0.848-1.726	0.379
>100	4.132	2.561-6.576	<0.001	2.475	1.421-4.264	0.007
no-exactly	2.761	1.995-3.823	<0.001	1.524	1.037-2.232	0.07
Total_nodes						
<8	Reference			Reference		
8~16	0.45	0.313-0.644	<0.001	0.337	0.217-0.518	<0.001
16-30	0.466	0.338-0.643	<0.001	0.247	0.163-0.372	<0.001
>30	0.329	0.215-0.494	<0.001	0.122	0.072-0.203	<0.001
Unkown	1.321	0.725-2.34	0.432	0.759	0.37-1.515	0.518

### Development and validation of a nomogram for predicting DM in early-onset GC

**Figure 4:**
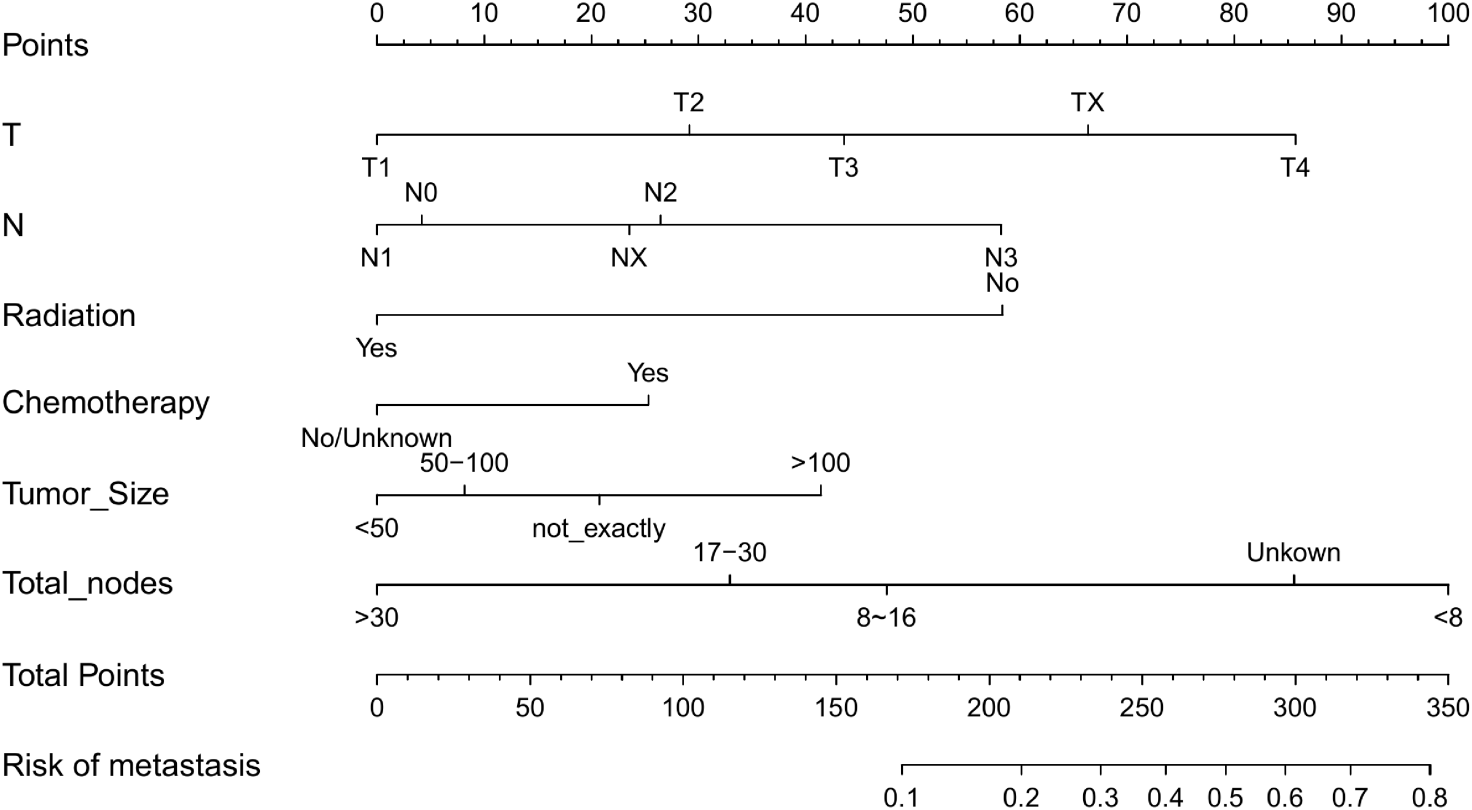
Nomogram for predicting DM in the training set of early-onset GC(C). DM, distant metastasis; GC, gastric cancer.

**Figure 5:**
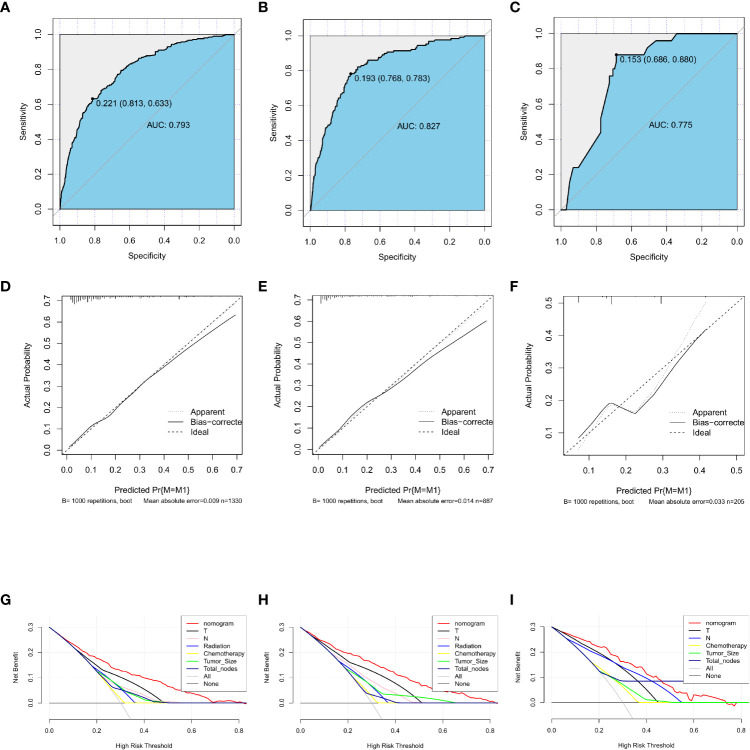
Training, testing, and validation sets of receiver operating characteristic (ROC) curves **(A–C)**, calibration curves **(D–F)**, and discriminant curve analyses (DCA) **(G–I)** for early-onset GC. GC, gastric cancer.

A nomogram was developed containing independent influencing factors identified through multiple logistic regression to predict DM in early-onset GC (**Figure 4**). Using the “pROC” package, we draw the ROC curves for the training and validation sets to test the model’s accuracy (**Figures 5A–C**). The AUC of our model in the training set was 0.827, while it was 0.793 and 0.775 in the internal test and external validation sets, indicating good prediction accuracy. Next, the calibration curves of the nomogram for evaluating the consistency of the expected and observed outcomes of DM revealed a high level of consistency between the nomogram-predicted overall survival and the actual result (**Figures 5D–F**). Finally, using the DCA analysis, we compared the nomogram model’s clinical value to that of various tumor clinicopathological characteristics (such as tumor size, T stage, and N stage) and found that the nomogram’s value was higher (red line in **Figures 5G–I**).

### Survival of patients with various nomogram scores

**Figure 6:**
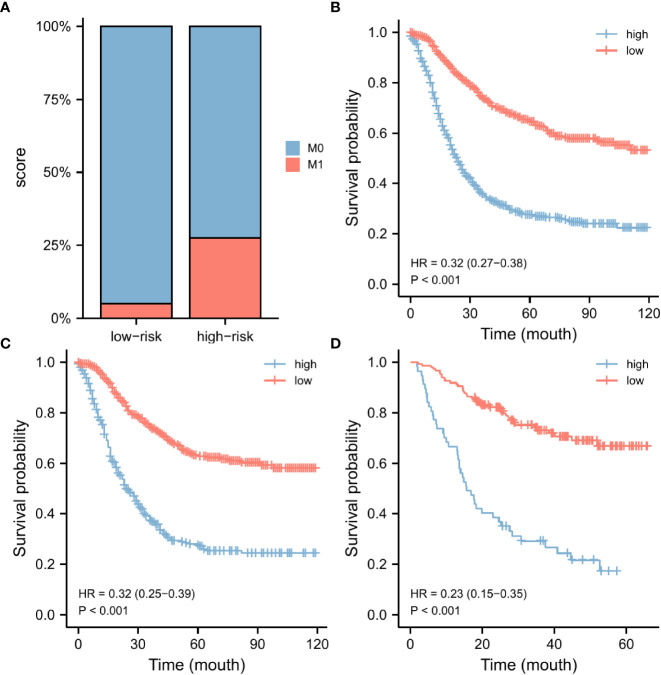
Nomogram risk score clinical application. According to the median risk score, our nomogram divides GC patients into two subgroups. There were various numbers of distant metastases in each subgroup **(A)**. The OS was evaluated in the training set **(B)**, the testing set **(C)**, and the external validation set **(D)**. GC, gastric cancer; OS, overall survival.

According to the nomogram, we extracted the patients from the training set again and then measured the risk scores for each GC patient. **Table 4** displays the risk scores associated with each clinicopathological variable. On the basis of the median of their total score, all patients were divided into two categories: low-risk (score < 174) and high-risk (score >= 174). We discovered that as the risk score increased, the likelihood of DM in patients with early-stage GC increased as well (**Figure 6A**). An increased risk score of DM in patients with early-onset GC was closely related to a worse prognosis, as shown by the Kaplan-Meier curve, which depicted the relationship between risk classification and prognosis (**Figure 6B**). The test and validation sets analysis confirmed the same result (**Figures 6C, D**).

**Table 4:** Score of every clinicopathological variable in our nomogram.

Clinicopathological variables	Nomogram score of distant metastasis
T stage	
T1	0
T2	29
T3	43
T4	86
Tx	66
N stage	
N0	4
N1	0
N2	27
N3	58
Nx	23
Radiation	
No	58
Yes	0
Chemotherapy	
No/Unknown	0
YES	25
Tumor size	
<50	0
50-100	8
>100	41
not_exactly	21
Total nodes	
<8	100
8~16	48
17-30	33
>30	0
Unkown	86

### Clinical and pathological differences in patients with different nomogram scores

**Figure 7:**
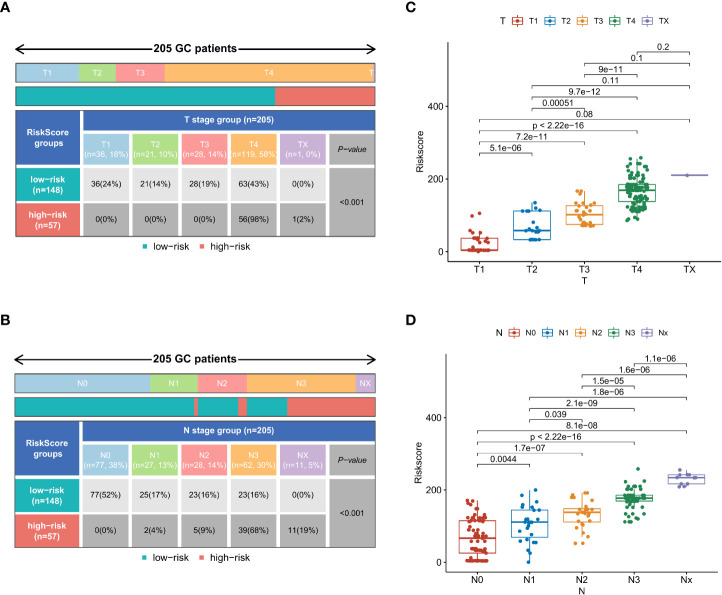
In the external validation set, the chi-square test results of risk stratification patients with different T stages **(A)** and N stages **(B)**. Comparison of risk scores between different subgroups in T-stage **(C)** and N-stage **(D)**.

To further explore the applicability of nomogram scores in clinical practice, we scored 205 patients with early-onset gastric cancer in our cohort according to the scoring principles of the training set. We analyzed the correlation between their scoring results and clinical-pathological factors. In the chi-square test of the case T stage, we found that patients with high scores were more likely to be in T3 and T4 stages (*p* < 0.001) (**Figure 7A**), and similarly, patients with N3 stage were more likely to obtain higher risk scores (**Figure 7B**). In Comparing different subgroups, higher T and N stages also have higher risk scores (**Figures 7C, D**). As an essential factor in the pathological characteristics of tumors, we used the same method to analyze the tumor size and the total number of lymph node dissections. The results showed that larger tumor diameter and fewer lymph node dissections could lead to higher scores (**Figures S2A, B**). In comparison, smaller tumor diameters and more lymph node dissections were often closely related to patients’ low-risk scores. (**Figures S2C, D**).

## Discussion

When compared to elderly patients, young patients are thought to have a poor prognosis for GC (10). Early-onset GC has a poor prognosis due to undifferentiated histology, unresectable lymphatic vascular infiltration, and late-stage diagnosis (11, 12). In reality, DM is the most important cause of therapeutic failure in the majority of malignant tumors. Most patients have locally advanced or DM when diagnosed with GC for the first time. The presence of DM has a direct impact on the treatment options available to patients as well as their overall prognosis. As a result, it is critical to comprehend the clinicopathological features of DM in GC. However, no research has been conducted on the risk factors for DM in GC under the age of 50. Many studies have shown that surgical resection combined with other systemic therapies could significantly improve the prognosis for patients with distantly metastatic GC (13). Therefore, we evaluated the potential risk factors for DM in early-onset GC and established a nomogram based on the clinical and pathological characteristics of the tumor to predict DM.

GC occurs at different rates in men and women. Men had a substantially higher incidence rate than women, and there was evidence that women had a favorable prognosis than men for gastric cancer (14). Interestingly, in our study, the metastatic rate of women with early-onset gastric cancer tends to be higher than that of men, because it was close to a significant value in the statistical calculation (*p* = 0.083). Gastric cancer is classified into two types based on its anatomical location: cardia GC and non-cardia GC. Non-cardia GC has a better prognosis and is frequently associated with Helicobacter pylori infection (15). However, our study found that the tumor location of patients with early-onset GC does not affect whether cancer has DM (*p*>0.05), which may need to be further confirmed in clinical studies at a more evidential level.

In addition, this study showed that in patients with early-onset GC, the size of the tumor, the total number of lymph nodes removed during surgery, the T4 and N3 tumor stages, and therapeutic interventions without radiation and chemotherapy were associated with the development of DM after surgery. Tumor grade, primary tumor diameter, T stage, and N stage were found to be predictors of synchronous DM in GC by Zikai Lin et al. (16). In accordance with the prior study, and we also included the size of the tumor, T stage, and N stage in our model. In our study, demographic factors such as age and gender were not defined as DM risk factors.

Furthermore, our study discovered a correlation between a larger tumor size and DM in early-onset GC. Previous research identified a tumor diameter greater than 2.5cm as an independent prognostic factor for patients with gastric signet ring cell carcinoma (17). Jingyu Chen et al. established a DM prediction model for superficial GC with no lymph node metastasis based on tumor diameter and other factors, they believed that a tumor larger than 2cm was a risk factor for DM of GC (18). Different target populations may result in different tumor diameters. We discovered that patients with tumor tissues larger than 10 cm had a greater risk of developing distant metastasis. Therefore, these patients require special attention. According to Qian Huang et al., early-onset GC is associated with diffuse Lauren type and poorly differentiated tumors (19). Surprisingly, after adjusting for other variables, the degree of tumor differentiation was eventually excluded from our prediction nomogram. We must recognize the clinical significance of tumor cell differentiation in early-onset GC completely because it has been defined as one of the most important features of GC patients in various studies.

Chemotherapy improved the survival and quality of life in patients with locally advanced gastric cancer (20). Patients with GC who received combined chemotherapy had a one-year median overall survival time (the survival time for Asian patients was often slightly longer). In contrast, patients receiving supportive treatment alone had a survival time of 3-4 months (21–23). It also explained that in our study, the majority of patients with DM and early-onset GC received chemotherapy. In contrast, randomized controlled trials have demonstrated that radiotherapy does not affect the overall survival of patients undergoing quality-assured D1 and D2 gastrectomy (24, 25). Patients with less than D1 + or D2 lymphadenectomy or R1 resection, on the other hand, can benefit from postoperative chemoradiotherapy (26). This is why our model considers radiotherapy to be a protective factor.

The nomogram is a useful and practical prediction tool that is widely used in research. Jie Cheng and colleagues developed a model to predict DM in patients with early-onset colorectal cancer (27). This approach was also widely used in the treatment of other cancers, such as lung (28) and esophageal cancer (29). However, most studies were unable to extend the risk model’s external validation. As a result, they cannot assess the model’s accuracy in actual clinical work. The prediction model developed in this study was evaluated using a similar internal validation set to the training set and the external validation set from two Chinese medical centers for GC, and we used the ROC curve, calibration curve, and DCA curve to verify our model’s accuracy, consistency, and clinical applicability. Finally, all patients were categorized as low-risk (score < 174) or high-risk (score >= 174) according to the risk score. The prognosis of the two groups differed significantly. The findings indicate that our model was trustworthy and could provide clinicians with additional information. Therefore, close supervision of DM should be considered for patients with early-onset GC who have a large tumor size, few lymph nodes discovered, tumor stages T4 or N3, and surgery combined with chemotherapy or without radiation. However, the limitation of our prediction model is that our follow-up sample size is not large enough. The accuracy and applicability of this model need to be verified in the follow-up data with a larger sample size.

In conclusion, our study found several independent risk factors for DM in people with GC who was younger than 50 years old. We also developed a nomogram that can predict DM in people with early-onset GC. The model’s prediction performance was good in both internal and external validation. As a result, they may be able to aid clinicians in predicting the progression of the disease and developing appropriate treatments.

## Data availability statement

The original contributions presented in the study are included in the article/**Supplementary Material**, further inquiries can be directed to the corresponding author/s.

## Ethics statement

Ethical approval was obtained from the Medical Ethics Committee of the Seventh affiliated Hospital of Sun Yat-sen University (No: KY-2020-024-01). All patient records and information were anonymized and deidentified prior to analysis.

## Author contributions

All authors contributed to the study’s conception and design. Y-LH and BB conceived the research. BB and G-FD performed material preparation, data collection, and analysis. BB and Y-MD wrote the first draft of the manuscript. Z-JH and X-YC participated in the revision of the article and the polishing of the language. Y-LH and C-HZ reviewed and edited the manuscript. All authors contributed to the article and approved the submitted version.
