# Supplementary material for: Retrospective analysis of risk factors for distant metastasis of early-onset gastric cancer during the perioperative period
Source: Front Oncol. 2023 Feb 3;13:1003977. doi: 10.3389/fonc.2023.1003977 (PMC9936415; doi:10.3389/fonc.2023.1003977)
Supplement: Supplementary file 1 [file DataSheet_1.docx]

**TABLE S1 Baseline clinical characteristics of patients in the external validation set**

|  | M0 | M1 | *p* |
| --- | --- | --- | --- |
| n | 166 | 39 |  |
| Age (%) |  |  | 0.682 |
| 20-29 | 8 (4.8) | 1 (2.6) |  |
| 30-39 | 46 (27.7) | 13 (33.3) |  |
| 40-49 | 112 (67.5) | 25 (64.1) |  |
| Sex = Male/Female (%) | 91/75 (54.8/45.2) | 16/23 (41.0/59.0) | 0.17 |
| Primary_Site (%) | |  | 0.014 |
| Lower | 79 (47.6) | 11 (28.2) |  |
| Middle | 57 (34.3) | 14 (35.9) |  |
| Upper | 18 (10.8) | 5 (12.8) |  |
| not_exactly | 12 (7.2) | 9 (23.1) |  |
| Grade (%) |  |  | 0.008 |
| Grade I | 7 (4.2) | 1 (2.6) |  |
| Grade II | 36 (21.7) | 6 (15.4) |  |
| Grade III | 119 (71.7) | 26 (66.7) |  |
| GX | 4 (2.4) | 6 (15.4) |  |
| Stage (%) |  |  | <0.001 |
| I | 44 (26.5) | 0 (0.0) |  |
| II | 41 (24.7) | 0 (0.0) |  |
| III | 78 (47.0) | 0 (0.0) |  |
| IV | 3 (1.8) | 39 (100.0) |  |
| T (%) |  |  | <0.001 |
| T1 | 36 (21.7) | 0 (0.0) |  |
| T2 | 21 (12.7) | 0 (0.0) |  |
| T3 | 26 (15.7) | 2 (5.3) |  |
| T4 | 83 (50.0) | 36 (94.7) |  |
| N (%) |  |  | <0.001 |
| N0 | 75 (45.2) | 2 (7.1) |  |
| N1 | 23 (13.9) | 4 (14.3) |  |
| N2 | 26 (15.7) | 2 (7.1) |  |
| N3 | 42 (25.3) | 20 (71.4) |  |
| M = M0/M1 (%) | 166/0 (100.0/0.0) | 0/39 (0.0/100.0) | <0.001 |
| Chemotherapy = No/Yes (%) | 64/102 (38.6/61.4) | 7/32 (17.9/82.1) | 0.025 |
| Tumor_Size (%) | |  | 0.017 |
| <50 | 95 (61.3) | 13 (36.1) |  |
| 50-100 | 53 (34.2) | 19 (52.8) |  |
| >100 | 7 (4.5) | 4 (11.1) |  |
| Total_nodes (%) | |  | <0.001 |
| <8 | 0 (0.0) | 11 (28.2) |  |
| 8~16 | 8 (4.8) | 2 (5.1) |  |
| 17-30 | 45 (27.1) | 10 (25.6) |  |
| >30 | 113 (68.1) | 16 (41.0) |  |


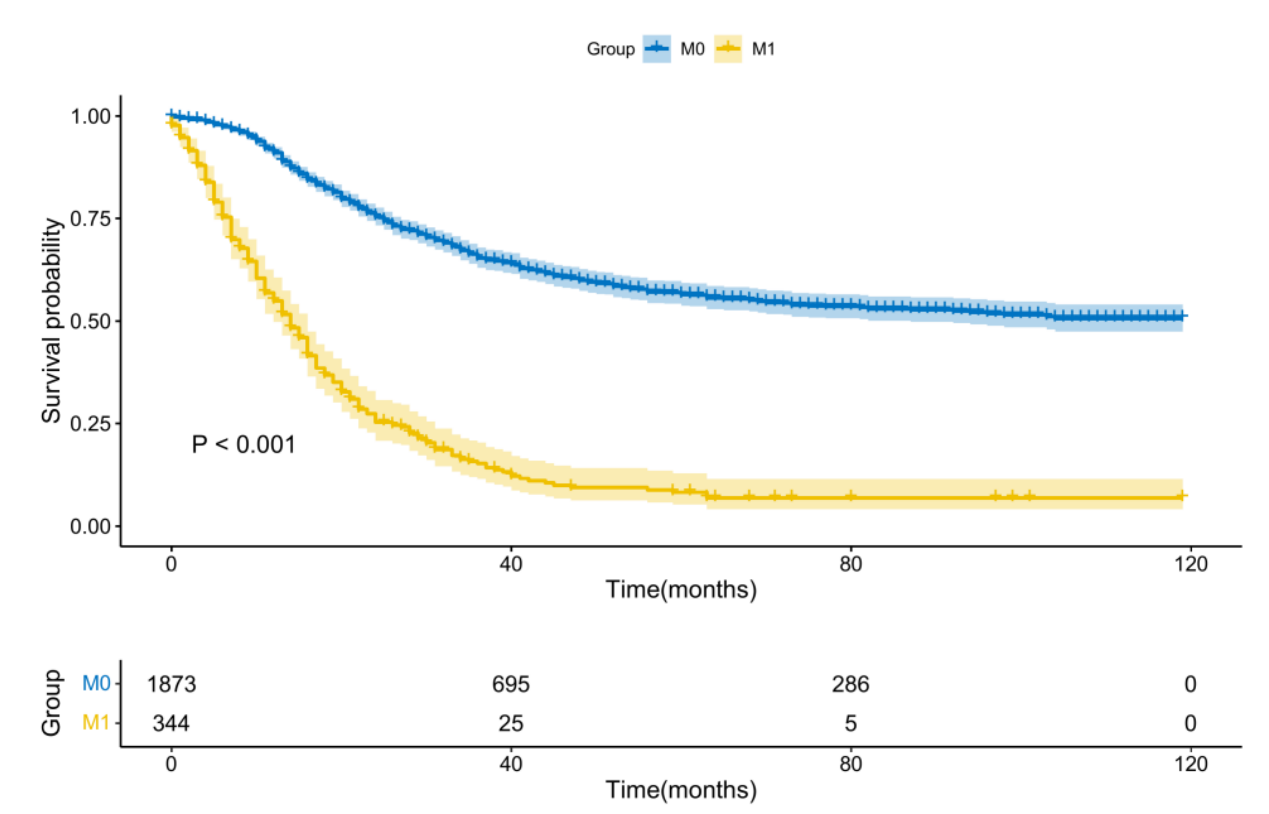


**Figure S1. KM curves of CSS for GC in the SEER data set. SEER, Surveillance, Epidemiology, and End Results database; CSS, cancer-specific survival; GC, gastric cancer**


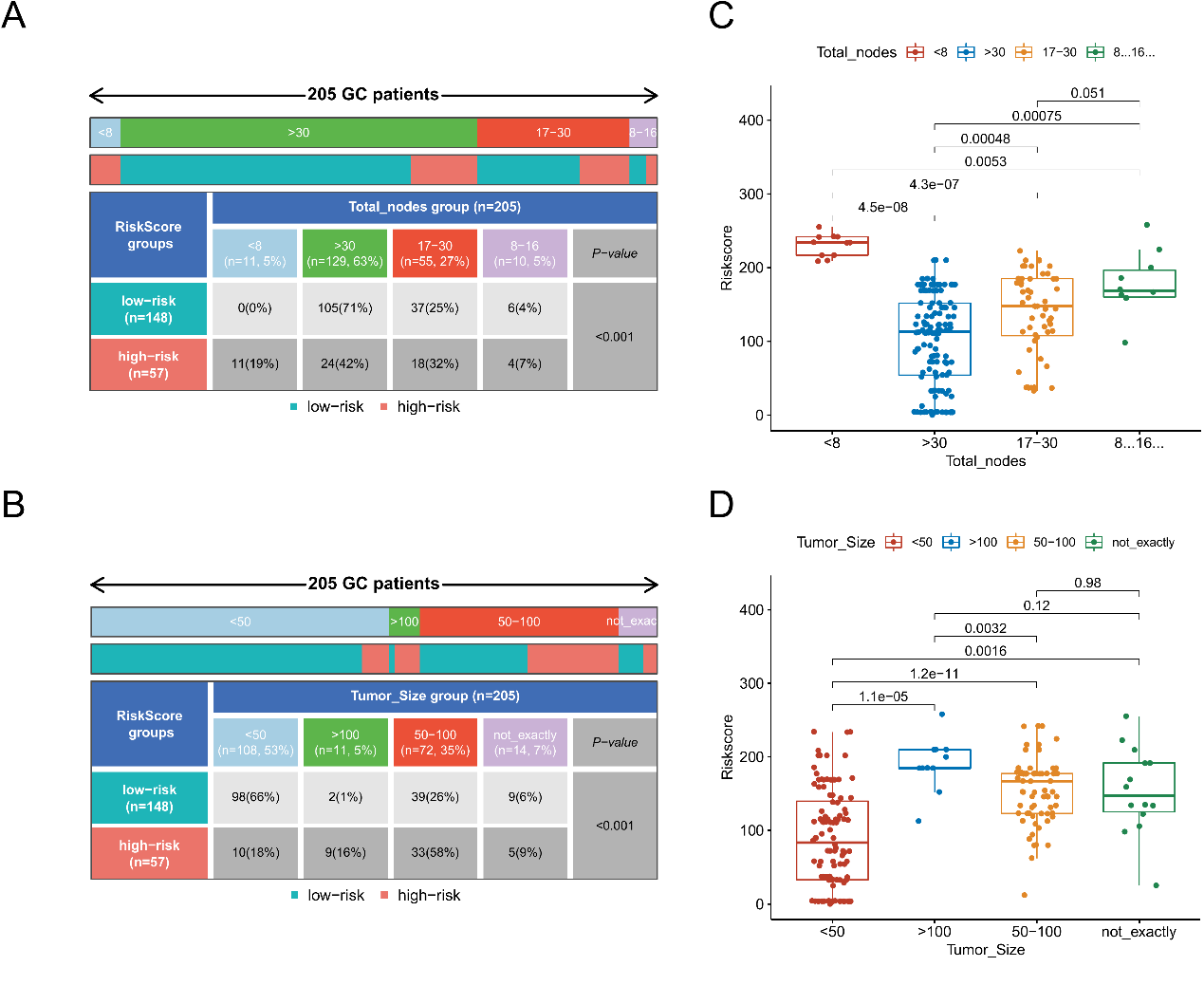


**Figure S2. In the external validation set, chi-square test results of risk stratification of patients with the different total number of lymph node dissections(A) and tumor sizes (B). Comparison of risk scores between different subgroups in patients with the different total number of lymph node dissections (C) and different tumor sizes (D).**
